# Olmesartan attenuates pressure-overload- or post-infarction-induced cardiac remodeling in mice

**DOI:** 10.18632/oncotarget.23628

**Published:** 2017-12-23

**Authors:** Qiancheng Wang, Zhenhuan Chen, Xiaobo Huang, Lin Chen, Baihe Chen, Yingqi Zhu, Shiping Cao, Wangjun Liao, Jianping Bin, Masafumi Kitakaze, Yulin Liao

**Affiliations:** ^1^ State Key Laboratory of Organ Failure Research, Department of Cardiology, Nanfang Hospital, Southern Medical University, Guangzhou 510515, China; ^2^ Department of Cardiology, Jiaozuo People's Hospital of Henan Province, Jiaozuo 454000, China; ^3^ Department of Oncology, Nanfang Hospital, Southern Medical University, Guangzhou 510515, China; ^4^ Cardiovascular Division of the Department of Medicine, National Cerebral and Cardiovascular Center, Suita, Osaka 565-8565, Japan

**Keywords:** periostin, olmesartan, myocardial hypertrophy, myocardial infarction, fibrosis

## Abstract

Either angiotensin converting enzyme inhibitor (ACEI) or angiotensin receptor 1 blocker (ARB) attenuates cardiac remodeling. However, the overall molecular modulation of the reversing remodeling process in response to the ACEI or ARB treatment is not yet well determined. In this study, we examined whether gene expressions are modulated by ACEI (temocapril), ARB (olmesartan) or both in a murine model with transverse aortic constriction (TAC) and confirm whether periostin is a target gene of olmesartan in mice with myocardial infarction (MI). We detected 109 genes that were significantly up-regulated in TAC mice and a majority of these were down-regulated in response to temocapril, olmesartan or their combination which significantly attenuated cardiac remodeling at one or four weeks. Real-time RT-PCR demonstrated that olmesartan, temocapril or their combination down-regulated the expression of periostin. In MI mice treated with olmesartan for 4 weeks, the left ventricular end-diastolic and systolic dimensions measured with echocardiography were lower, whereas maximum rate of rise and fall rate of LV pressure (±dp/dt max) were greater, and Azan-staining cardiac fibrotic area was smaller. Furthermore, periostin was upregulated in response to MI, whereas olmesartan blocked this upregulation. Post-MI fibrosis was smaller in periostin knockout adult mice than in wildtype mice, while glycogen synthase kinase 3β was increased and cyclin D1 was decreased in periostin knockout mice. These findings indicate that periostin is a target gene of ARB and olmesartan reverses cardiac remodeling at least partially through the downregulation of periostin.

## INTRODUCTION

It has been widely recognized that angiotensin II receptor blockers (ARBs) or angiotensin-converting enzyme inhibitors (ACEIs) exert beneficial effects in term of mortality and morbidity in patients with chronic heart failure, and also favorably modulate the left ventricular remodeling process [1–3]. These effects of reversing remodeling include a decrease in cardiac hypertrophy [4, 5], and improvements in the systolic or diastolic cardiac function [6, 7]. However, the overall gene modulation of the reverse remodeling process in response to ACEI and/or ARB is not yet well understood, although several clinical studies [8, 9] support the view that gene expression profiling may ultimately enhance diagnostic precision and help guide or monitor therapy. Previous investigations following ARB and ACEI treatment have focused on the expression of various genes of interest, and found that several genes were down-regulated, such as brain natriuretic peptides [4], collagen [7], ACE, transforming growth factor (TGF)-beta1 [10], and calpain mRNAs [11]. Such reports have given us insight into some of the molecular mechanisms that may be involved in drug-mediated myocardial recovery. Gene expression profiling during cardiac hypertrophy and regression of cardiac hypertrophy has been investigated [12], but global gene expression has not yet been examined in parallel with regression of cardiac hypertrophy in response to pharmacological intervention such as with ACEI or ARB in pressure-overloaded heart.

By gene chip screening, we confirmed that pharmacological treatment with temocapril (also called CS622), olmesartan (also called CS866) or their combination significantly blocked the upregulation of periostin. Periostin plays an important role during cardiac development and in the epithelial-mesenchymal transition [13], and it is also closely associated with cardiovascular diseases such as dilated cardiomyopathy and myocardial infarction (MI) [14]. Several studies have demonstrated function for periostin in regeneration of tissues including the myocardium [15–18]. However, it is unclear whether periostin is also a target gene of ARB for attenuating post-MI remodeling.

This study is to investigate the global gene expression in parallel with regression of cardiac hypertrophy in response to treatment with temocapril or olmesartan in mice with transverse aortic constriction (TAC), and to confirm whether periostin is a target gene of olmesartan in mice with post-MI cardiac remodeling.

## RESULTS

### Temocapril, olmesartan or their combination attenuates myocardial hypertrophy

**Figure 1:**
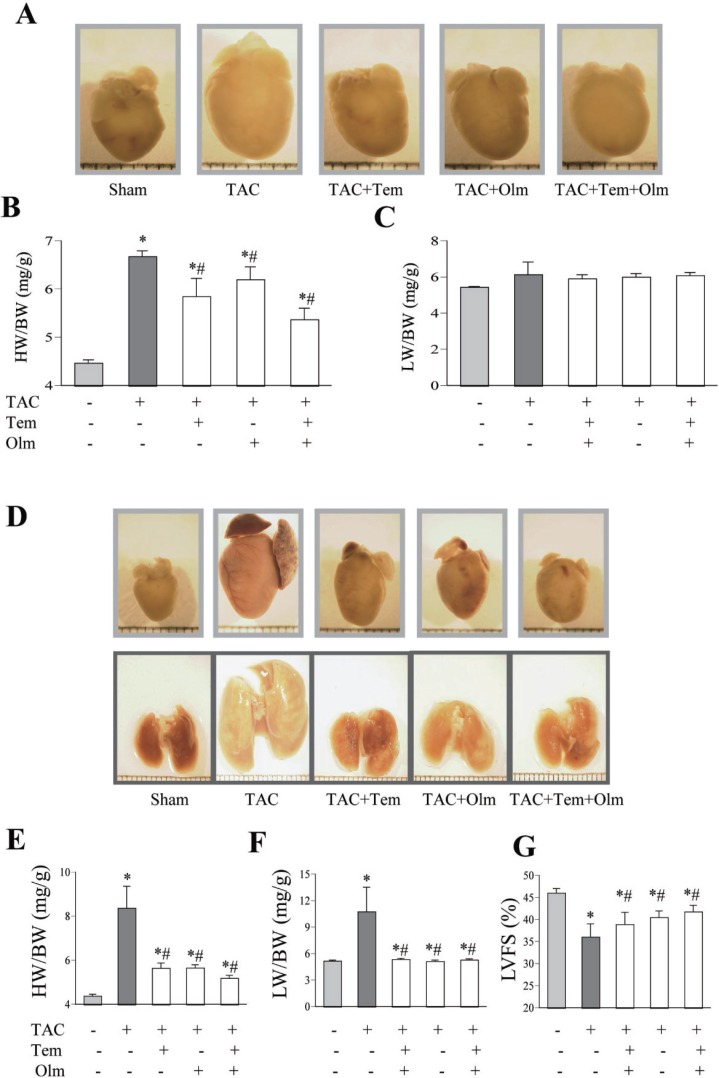
Antihypertrophic effect of temocapril, olmesartan or their combination (**A**) Representative pictures of whole heart at 1 week after TAC or sham operation with or without drug-treatment. (**B**) Heart weight to body weight ratio (HW/BW) and (**C**) lung weight to body weight ratio (LW/BW) at 1 week after TAC, treatment with or without temocapril (Tem), olmesartan (Olm) or their combination. ^*^P < 0.05 vs. sham group, ^#^*P <* 0.05 vs. TAC group in panel B and C. (**D**) Representative pictures of whole heart and lung at 4 weeks after TAC or sham operation with or without drug-treatment. (**E**) HW/BW ratio and (**F**) LW/BW ratio at 4 weeks after TAC, treatment with or without temocapril, olmesartan or their combination. (**G**) Left ventricular systolic function, quantified by left ventricle fractional shortening (LVFS) at 4 weeks after TAC in five groups. ^*^P < 0.05 vs. sham group, ^#^*P <* 0.05 vs. TAC group in panel E, F and G.

As shown in Figure 1A–1B, TAC procedure increased the heart weight to body weight ratio (HW/BW) at 1 week in the TAC group relative to the sham-operated group, and the treatment with olmesartan, temocapril or their combination reduced it after TAC (Figure 1B). While no significant difference of lung weight to body weight ratio (LW/BW) was noted between the drug-treated and vehicle-treated mice (Figure 1C). At 4 weeks after TAC, both HW/BW and LW/BW were greater in the TAC group than in sham group, but these parameters were significantly lower in drug-treated TAC groups than in the TAC alone group (Figure 1D–1F). Furthermore, the left ventricle shortening fraction (LVFS) at 4 weeks after TAC was lower in TAC group than in sham group (Figure 1G), whereas treatment with olmesartan, temocapril or their combination improved LVFS (Figure 1G). Although the combination therapy showed a tendency to attenuate cardiac hypertrophy to a greater extent than in the single olmesartan or temocapril group, no statistically significant difference was observed. We noticed that the degree of cardiac hypertrophy at 1 and 4 weeks’ time points in 3 drug-treated groups is similar, while in vehicle treated TAC mice, the HW/BW was increased time-dependently, suggesting that those drugs effectively prevented the progress of cardiac hypertrophy.

### Gene expression profiles in a time-course and the upregulated genes in response to TAC

**Figure 2:**
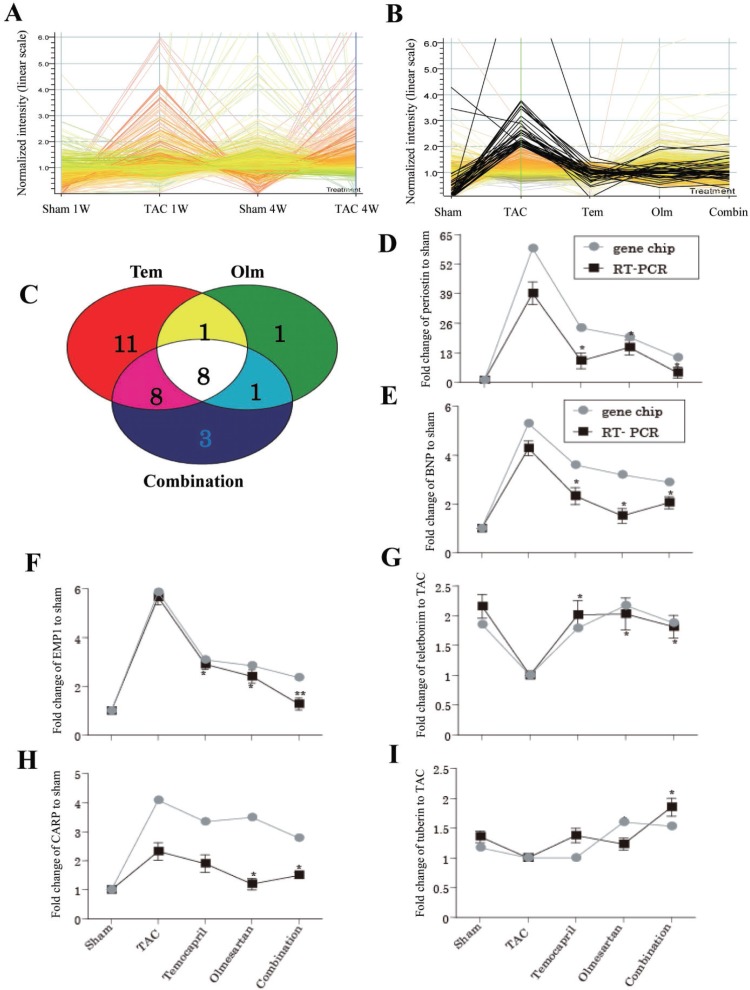
Gene expression patterns in response to TAC and drug treatment (**A**) A time course of global gene expression at 1 and 4 weeks’ time points. As shown by the red lines, expression pattern in response to TAC at 1 and 4 weeks is similar. (**B**) The high expression genes with more than 2 folds upregulation in average difference relative to control signal in TAC group demonstrated a tendency of downregulation in sham and 3 drug-treated groups. (**C**) Among the upregulated 109 genes in TAC relative to sham, 8 co-regulated genes was identified. (**D**–**I**) Validation of the microarray results by using quantitative PCR. BNP: natriuretic peptide precursor type B, EMP1: epithelial membrane protein 1, CARP: cardiac ankyrin repeat protein mRNA. Combin or combination: combination with temocapril (Tem) and olmesartan (Olm). ^*^P < 0.05 vs, ^**^P < 0.01. TAC group in panel D–I.

Since both one and four weeks of TAC can induce cardiac hypertrophy, their gene expression pattern may be similar. To verify this speculation, we performed cDNA microarray analysis. Indeed, as shown in Figure 2A, the pattern of the expression of the upregulated genes shown with red lines was similar in TAC mice at 1 and 4 weeks’ time point, although the levels of expression were different for a set of genes such as B-type natrium peptide (BNP). Considering these results, we examined the effect of drug-treatment on the gene expression profile in either one or four weeks’ time point. As shown in Figure 2B, the high expression genes with more than 2 folds upregulation relative to control signal in TAC group demonstrated a tendency of downregulation in mice treated with temocapril, olmesartan or their combination. We have detected 109 transcripts with a more than 2 folds increase. Among the 109 genes, 76 genes listed in Table 1 belonged to different functional catalogues. The significant upregulated genes in vehicle-treated TAC mice relative to sham were downregulated by the treatment with temocapril, olmesartan or their combination.

**Table 1:** List of genes with altered expression in response to hypertrophy and pharmacological intervention

Gene name	Accession No	Sham	TAC	Temocapril	Olmesartan	Comb
M.musculus col8a1 gene, exon 1	X66976	0.001	3.718	**0.675**	**1.308**	**1.225**
Fibrillin 1	L29454	0.001	3.014	0.663	1.000	1.399
Complement component 1, q subcomponent, c polypeptide	X66295	0.001	2.248	0.430	**1.000**	1.574
Epithelial membrane protein 3	U87948	0.001	2.021	**0.698**	1.161	1.657
Procollagen, type I, alpha 2	X58251	0.003	3.586	1.271	**0.802**	**1.000**
Fibulin 2	X75285	0.008	2.784	0.905	0.981	1.308
Mus musculus Plp2 mRNA for proteolipid protein 2	AB031292	0.007	1.390	0.842	0.949	1.086
Follistatin-like	M91380	0.028	3.174	**1.052**	1.070	**0.920**
Complement component 1 inhibitor	AF010254	0.020	1.769	0.707	1.170	1.000
Mus musculus osf-2 mRNA for osteoblast specific factor 2	D13664	0.059	3.459	**1.408**	1.171	**0.644**
Mus musculus alpha-1 type I procollagen mRNA	U03419	0.065	3.764	**1.051**	**0.798**	**1.031**
Cysteine rich intestinal protein	M13018	0.048	2.025	0.869	1.880	2.098
Mouse calpactin I heavy chain (p36) mRNA	M14044	0.087	2.027	0.952	0.943	1.029
Mouse fibronectin (FN) mRNA	M18194	0.161	3.564	**0.938**	**1.024**	**1.000**
Interferon beta, fibroblast	V00755	0.121	2.523	**1.081**	0.607	**1.015**
Procollagen, type III, alpha 1	X52046	0.143	2.257	**1.036**	0.790	**0.802**
Mus musculus endothelial monocyte-activating polypeptide I mRNA	U41341	0.154	2.153	**0.923**	1.104	1.182
Mouse mRNA for cysteine-rich glycoprotein SPARC	X04017	0.203	2.071	**0.824**	1.065	**1.027**
Retinol binding protein 1, cellular	X60367	0.283	2.551	**0.807**	**1.191**	**1.000**
Mouse complement C1q B chain mRNA	M22531	0.215	1.907	**0.797**	1.080	1.264
Actin, alpha 1, skeletal muscle	M12347	0.166	1.467	0.883	1.176	1.036
Procollagen, type IV, alpha 1	M15832	0.230	1.914	0.828	1.048	1.080
Natriuretic peptide precursor type B	D16497	0.337	1.789	1.218	1.079	0.973
Epithelial membrane protein 1	X98471	0.377	1.974	1.038	0.959	0.889
Complement component 1, q subcomponent, alpha polypeptide	X58861	0.318	1.443	0.865	0.945	1.015
M.musculus mRNA for PGI (biglycan)	X53928	0.348	1.557	1.000	0.919	0.821
Mus musculus suppressor of cytokine signalling-2 (SOCS-2) mRNA	U88327	0.355	1.571	**0.704**	1.359	0.999
Mus musculus cardiac ankyrin repeat protein MCARP mRNA	AF041847	0.324	1.322	1.084	1.132	0.901
Procollagen, type VI, alpha 2	Z18272	0.489	1.973	1.000	1.077	1.067
Mus musculus protein inhibitor of nitric oxide synthase (PIN) mRNA	AF020185	0.533	2.076	1.079	1.124	**0.911**
Murine MLC1F/MLC3F gene for myosin alkali light chain	X12973	0.483	1.861	1.035	0.735	0.786
Clusterin	D14077	0.594	2.227	**1.047**	0.976	**0.921**
Mus musculus CDC45-related protein (Cdc45) mRNA	AF098068	0.367	1.368	**0.503**	1.000	1.463
Mouse procollagen type V alpha 2 (Col5a-2) mRNA	L02918	0.668	2.446	**1.081**	0.870	**0.747**
Mus musculus thymic shared antigen-1 (TSA-1) gene	U47737	0.344	1.258	0.763	1.032	1.062
Calmodulin	M19381	0.401	1.457	0.870	1.187	1.233
Lumican	AF013262	0.478	1.687	1.000	0.987	0.730
Cytotoxic T lymphocyte-associated protein 2 alpha	X15591	0.476	1.650	1.228	1.151	0.788
Mus musculus LIM domain transcription factor LMO4 mRNA	AF074600	0.412	1.357	0.738	0.962	1.304
Cofilin 1, non-muscle	D00472	0.572	1.862	0.998	1.019	1.095
Natriuretic peptide precursor type A	K02781	0.623	2.005	**0.986**	1.068	0.879
Mus musculus mRNA for cathepsin S, partial	AJ223208	0.687	2.178	**1.032**	0.994	**0.741**
Hypoxia inducible factor 1, alpha subunit	AF003695	0.420	1.303	0.745	1.081	1.181
Mouse mCyP-S1 mRNA for cyclophilin CyP-S1	X58990	0.432	1.323	0.871	1.055	1.020
Apolipoprotein E	D00466	0.542	1.630	0.840	1.229	0.977
Islr(immunoglobulin superfamily containing leucine-rich repeat) mRNA	AB024538	0.522	1.500	0.993	1.124	0.799
Mouse mRNA for prothymosin alpha	X56135	0.506	1.381	0.859	1.200	0.855
Matrix gamma-carboxyglutamate (gla) protein	D00613	0.694	1.888	1.108	1.152	**0.934**
Transcription factor 4	U16322	0.568	1.519	0.919	1.254	0.753
Transgelin	Z68618	0.845	2.244	**1.105**	**0.768**	1.172
Calcyclin	X66449	0.514	1.333	0.816	1.020	1.058
Mus musculus mRNA for sid23p	AB025406	0.488	1.248	0.914	1.013	1.382
P glycoprotein 2	J03398	0.394	1.000	0.748	1.523	1.242
Mouse 3’ mRNA for beta-galactoside specific lectin (14kDa)	X15986	0.607	1.532	0.888	1.000	1.169
Murine mRNA for J1 protein, yeast ribosomal protein L3 homologue	Y00225	0.508	1.266	0.859	1.079	1.063
Mouse (with short incubation period) prion protein (PRNP) gene	M18070	0.502	1.234	0.813	1.234	1.019
Lethal giant larvae homolog	D16141	0.514	1.261	1.197	0.944	0.606
Cd63 antigen	D16432	0.487	1.193	0.707	1.169	1.001
Glutathione peroxidase 3	U13705	0.712	1.729	1.064	0.847	0.980
Tubulin alpha1	M28729	0.615	1.487	0.975	0.966	1.192
Insulin-like growth factor binding protein 5	L12447	0.433	1.043	0.674	1.209	1.120
Glutamate receptor, ionotropic, NMDA2C (epsilon 3)	L35029	0.792	1.900	1.327	0.851	**0.500**
Core binding factor beta	L03279	0.649	1.510	0.697	1.180	1.257
Murine mRNA for replacement variant histone H3.3	X13605	0.476	1.105	0.608	1.124	1.046
Prothymosin beta 4	U38967	0.661	1.520	0.986	1.131	1.000
Hypoxia inducible factor 1, alpha subunit	Y09085	0.556	1.253	0.929	1.050	0.948
Procollagen, type VI, alpha 1	X66405	0.622	1.402	1.000	0.861	0.965
Mouse eEF-Tu gene encoding elongation factor Tu	M17878	0.640	1.425	1.000	0.957	0.871
Benzodiazepine receptor, peripheral	D21207	0.765	1.673	1.000	0.977	1.029
Mouse cytochrome beta-558 mRNA	M31775	0.607	1.324	0.785	1.194	1.110
Mus musculus APC-binding protein EB1 homolog mRNA	U51196	0.461	1.000	0.703	1.279	1.232
Mouse E46 mRNA for E46 protein	X61506	0.565	1.216	1.000	0.948	1.162
Mouse mRNA for fibronectin receptor beta-chain (VLA5-homolog.)	X15202	0.629	1.344	1.003	1.129	0.922
Hypoxanthine guanine phosphoribosyl transferase	K01515	0.550	1.137	1.029	0.869	0.788
Actin, gamma 2, smooth muscle, enteric	U20365	0.563	1.150	1.107	0.973	1.033
Mus musculus Ap-3 complex beta3A subunit (Ap3b1) mRNA	AF103809	0.558	1.134	0.905	1.090	0.875

Gene expression levels were expressed as Means of 2 samples. Data significantly different relative to TAC are shown in bold font. TAC: transverse aortic constriction; Comb: temocapril plus olmesartan.

### Genes with altered expression during the treatment with ACEI and/or ARB and its validation

Among the 109 up-regulated genes in the TAC group, temocapril, olmesartan and their combination therapy down-regulated 28, 11 and 20 genes by more than twofold relative to the TAC group, respectively (Figure 2C). Of these, 8 genes were down regulated similarly in all of 3 pharmacologically treated groups. Compared with the TAC group, 11 genes such as telethonin, tuberous sclerosis 2, and prohibitin were significantly upregulated in at least one of the 3 drug treatment groups (Table 2).

**Table 2:** List of genes up-regulated by pharmacological intervention

Gene name	Accession No	Sham	TAC	Temocapril	Olmesartan	Comb
Mus musculus telethonin genomic sequence Mus musculus mCAC gene	AJ223855	1.034	0.558	**1.000**	**1.213**	1.049
	AB017112	0.952	0.763	0.911	**1.219**	1.033
Mus musculus ATP-specific succinyl-CoA synthetase beta subunit (Scs) mRNA	AF058955	1.139	0.949	0.940	**1.472**	0.845
Nicotinamide nucleotide transhydrogenase	Z49204	1.089	0.825	1.000	**1.268**	1.049
Mus musculus S3-12 mRNA	AF064748	1.218	0.788	0.750	**1.195**	0.914
Prohibitin	X78682	0.937	0.769	0.712	**1.162**	**1.418**
Mus musculus aldehyde dehydrogenase (ALDH2) mRNA,	U07235	0.995	0.705	0.776	1.815	**1.515**
Solute carrier family 4 (anion exchanger), member 3	M28383	1.065	0.755	0.888	1.538	**1.550**
Mouse mRNA for protein with homology to transition protein 2 (TP2)	X17069	1.027	0.686	0.678	1.364	**1.080**
Tuberous sclerosis 2	U37775	1.000	0.852	0.850	1.360	**1.302**
Glutathione S-transferase, pi 2	X53451	1.000	0.868	0.734	1.324	**1.311**

Gene expression levels were expressed as Means of 2 samples. Data significantly different relative to TAC are shown in bold font. Comb: Temocapril plus Olmesartan.

Six genes were selected for real-time PCR. Figure 2D–2I showed the fold-change of six candidate genes in all the experimental groups. Myocardial BNP, epithelial membrane protein 1 (EMP1), ankyrin repeat domain 1/cardiac ankyrin repeat protein (Ankrd1/CARP) and periostin genes showed significantly elevated expressions in the TAC group relative to the sham-operated group. Interestingly, pharmacological intervention with temocapril, olmesartan or their combination effectively attenuated the extent of elevation of those gene expressions, whereas those of telethonine and tuberin were upregulated in response to the drug treatment.

### Effect of ankrd 1 and periostin on myocyte hypertrophy or cardiac remodeling

**Figure 3:**
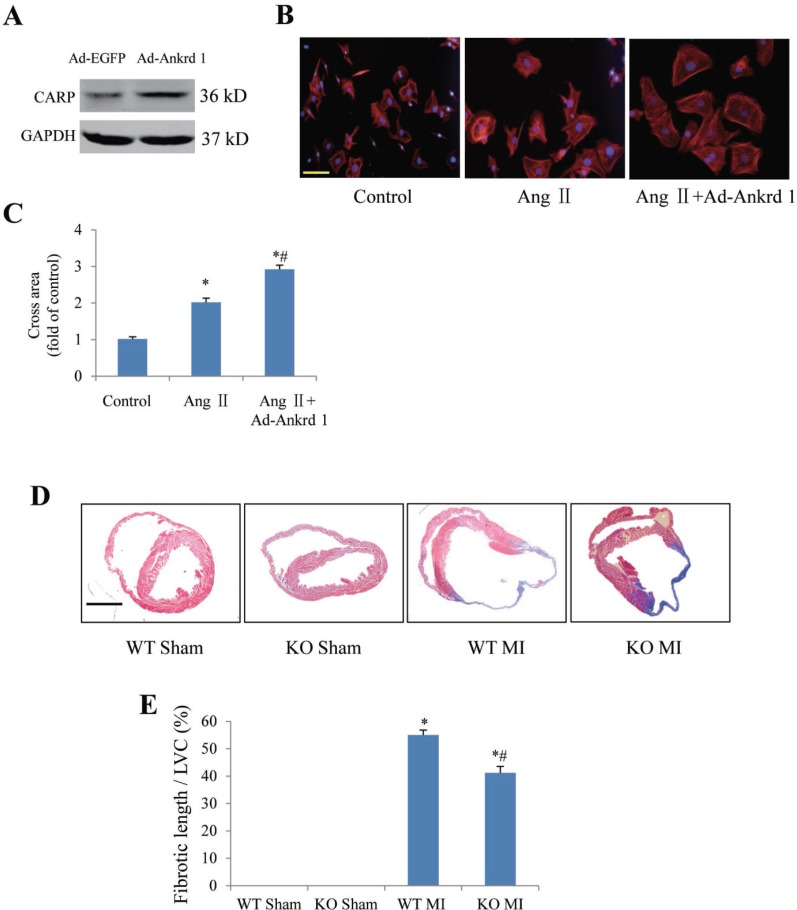
Confirmation of ankrd1 and periostin involving in myocyte hypertrophy or post-MI cardiac remodeling by using approaches of gain and loss of function (**A**) Western blotting confirmation of ankrd1 overexpression with adenovirus carrying ankrd1 plasmid (Ad-Ankrd1) in neonatal rat cardiomyocytes. (**B**) Representative pictures showing cross area of cardiomyocytes in response to angiotensin II (Ang II) stimulation in the presence/absence of ankrd1 overexpression. Scale bar = 50 µm. (**C**) Ankrd1 overexpression enhanced Ang II-induced increase of cardiomyocyte area. ^*^P < 0.05 vs. control group, ^#^*P <* 0.05 vs. Ang II alone group, experiments were repeated for 3 times. (**D**) Representative pictures of cardiac cross section with Masson staining 4 weeks after myocardial infarction (MI). Scale bar = 2 mm. (**E**) Periostin knockout (KO) attenuated post-MI fibrosis. ^*^P < 0.05 vs. sham group, ^#^*P <* 0.05 vs. MI alone group, *n* = 6 in each group. WT, wildtype; LVC, left ventricular circumference.

We further confirmed our microarray findings by using gain and loss of function approaches for interest genes ankrd 1 and periostin. Ankrd1 was overexpressed in neonatal rat cardiomyocytes with adenovirus (Figure 3A). Phalloidine staining indicated that overexpression of ankrd 1 enhanced angiotensin II (Ang II)-induced increase of cardiomyocyte area (Figure 3B, 3C). In mice with MI for 4 weeks, masson’s trichrome staining indicated that myocardial fibrosis area (fibrotic length/LV circumference) was markedly lower in periostin knockout mice than in wildtype mice (Figure 3D and 3E).

### Olmesartan alleviates post-MI dysfunction in mice

**Figure 4:**
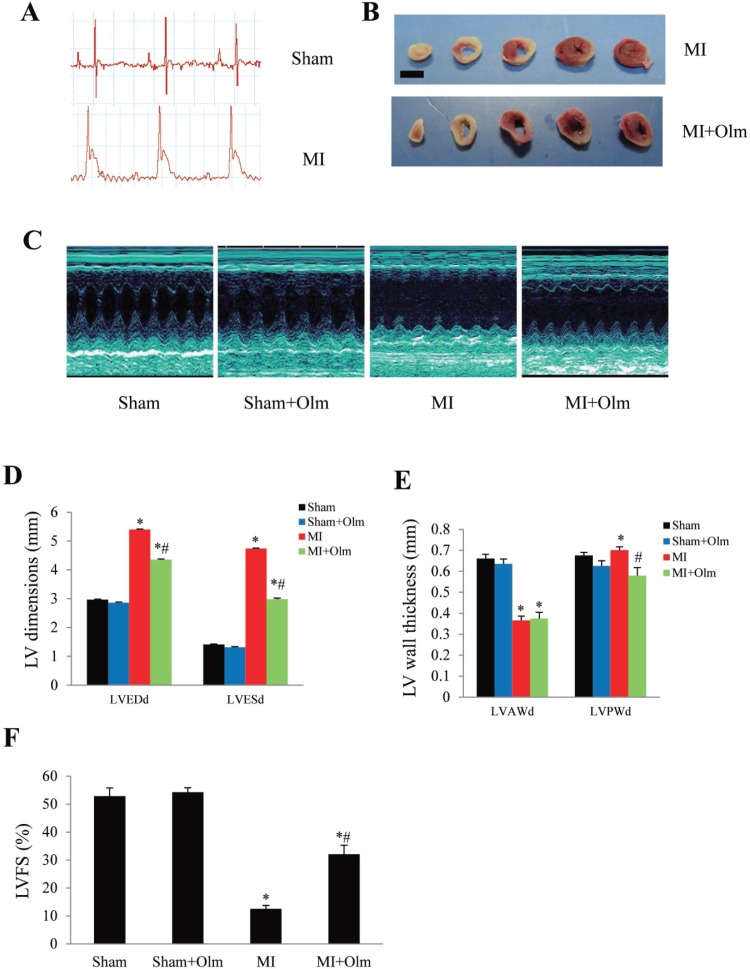
Treatment of olmesartan attenuates left ventricular dysfunction at 4 weeks after MI (**A**) ST segment elevation in electrocardiogram monitoring showed an MI model was successful. (**B**) TTC staining in mice 24 h after MI showed the infarcted area (fibrotic length) was similar in olmesartan (Olm)-treated and untreated MI groups. The scale bar is 5 mm. (**C**) Representative images of M-mode echocardiography. (**D**) Results of echocardiographic left ventricular dimensions. LVEDd, left ventricular end-diastolic diameter; LVESd, left ventricular end-systolic diameter. (**E**) Results of echocardiographic left ventricular wall thickness. LVAWd: left ventricular anterior wall thickness; LVPWd: left ventricular posterior wall thickness. (**F**) Results of echocardiographic left ventricular fractional shortening (LVFS). ^*^P < 0.05 vs. their corresponding sham group, ^#^*P <* 0.05 vs. MI group in panel D–F.

To investigate the effect of olmesartan on post-infarction remodeling, we created an MI model in C57/BL6 mice (Figure 4A). The infarcted area was similar between drug-treated and vehicle-treated groups 24 hours after MI (Figure 4B). We found that left ventricular end-diastolic dimension (LVEDd) and left ventricular end-systolic dimension (LVESd) were significantly increased at 4 weeks after MI, while MI mice treated with olmesartan have smaller LV dimensions (Figure 4D). Furthermore, the left ventricular anterior wall thickness (LVAWd) was decreased in response to MI, and no significant difference was found between drug-treated and vehicle-treated MI groups (Figure 4E). The left ventricular posterior wall thickness (LVPWd) was increased in MI mice, whereas olmesartan blocked the increase of LVPWd (Figure 4E). The LVFS was greater in olmesartan-treated MI mice than in vehicle-treated MI mice (Figure 4F).

### Olmesartan improves post-MI left ventricular hemodynamic

**Figure 5:**
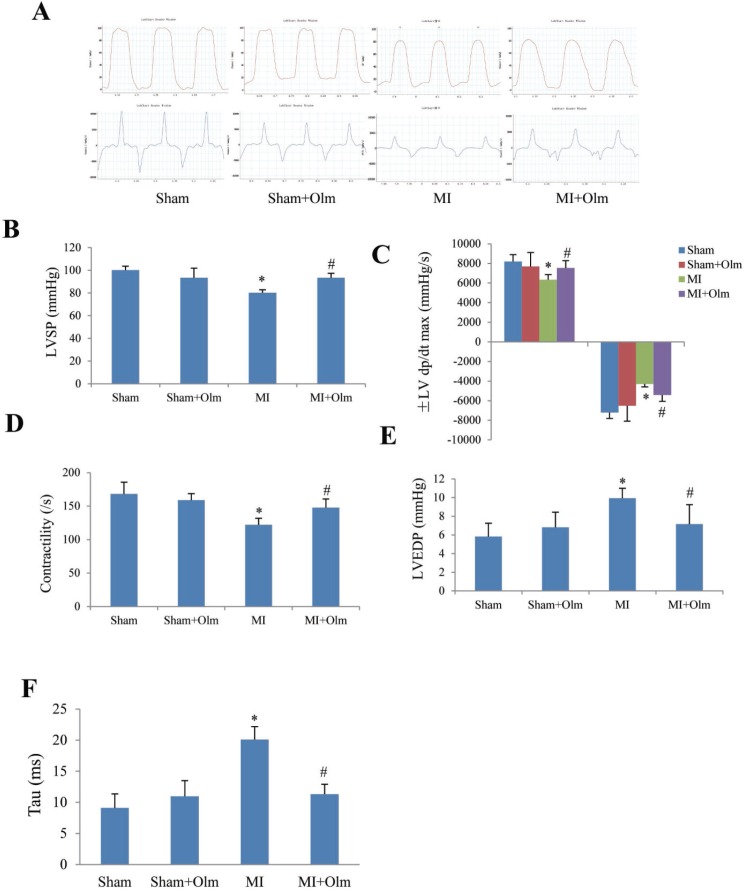
Olmesartan (Olm) treatment improves left ventricular hemodynamic at 4 weeks after myocardial infarction (MI) (**A**) Examples of left ventricular pressure curve recording. (**B**) Left ventricular systolic pressure (LVSP). (**C**) Maximum rates of left ventricular rising and descending (dp/dt max, dp/dt min). (**D**) Left ventricular contractility. (**E**) Left ventricular end-diastolic pressure (LVEDP). (**F**) The exponential time constant of left ventricular relaxation (tau). ^*^P < 0.05 vs. sham group, ^#^*P <* 0.05 vs. MI group in panel D–F.

The LV hemodynamic assessment (Figure 5A) showed that MI for 4 weeks resulted in a significant reduction of systolic pressure (LVSP) (Figure 5B), ± dp/dt max (Figure 5C) and contractility (Figure 5D) as well as a significant increase of LV end-diastolic pressure (LVEDP) (Figure 5E) and exponential time constant of relaxation (tau) (Figure 5F) when compared with the corresponding sham group. Whereas, olmesartan treatment in MI mice blocked these changes (Figure 5B–5F).

### Olmesartan attenuates post-infarction myocardial remodeling and pulmonary congestion

**Figure 6:**
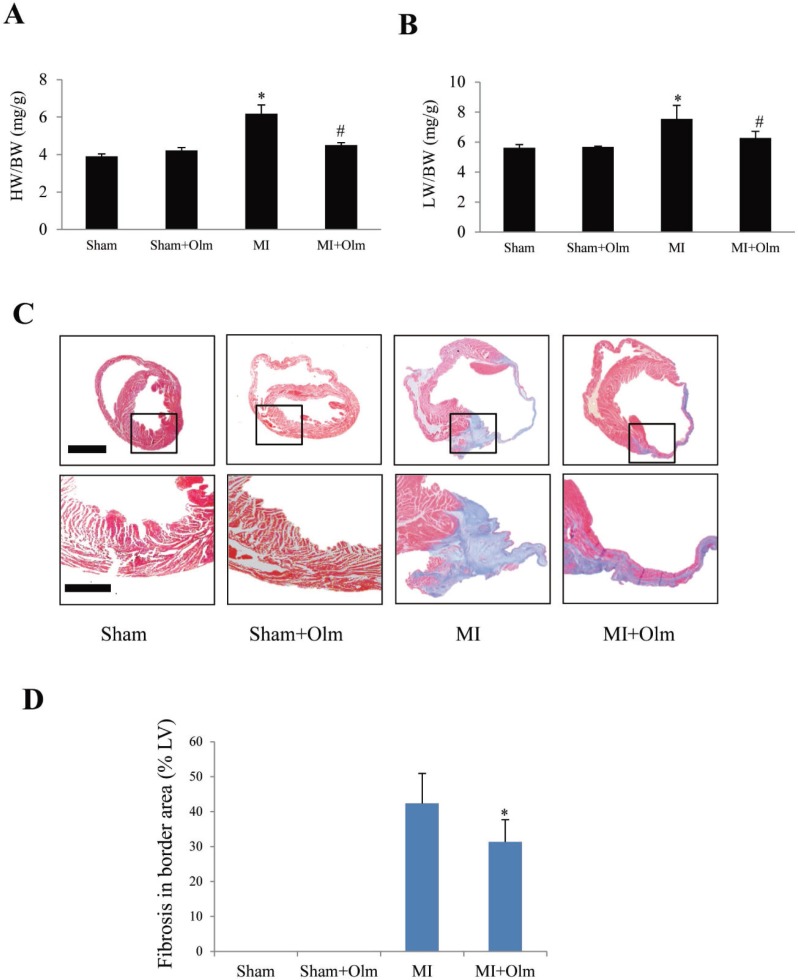
Olmesartan alleviates post-infarction remodeling at 4 weeks (**A**) Heart weight/body weight (HW/BW) at 4 weeks after MI with or without olmesartan (Olm) treatment. (**B**) Lung weight/BW (LW/BW) increased in mice subjected to MI and decreased by olmesartan treatment. ^*^P < 0.05 vs. sham group, ^#^*P <* 0.05 vs. MI group. (**C**) Masson’s trichrome staining of hearts obtained at 4 weeks after MI. The lower panels show magnifications of the regions indicated by black boxes in the upper panels. The scale bar is 1 mm in the upper panel and 100 μm in the lower panel. (**D**) Quantitation of old myocardial infarction area (Masson’s trichrome positive staining length of LV/LV circle). ^*^ *P <* 0.05 vs. MI group. IA: infarct area).

At 4 weeks after MI, HW/BW (Figure 6A) and LW/BW (Figure 6B) were larger in the MI group than in sham group, whereas these indices were smaller in olmesartan-treated MI mice than in vehicle-treated MI group (Figure 6A and 6B). Masson’s trichrome staining indicated that myocardial fibrosis area (fibrotic length/LV circumference) was markedly larger in MI mice than in olmesartan-treated MI mice (Figure 6C and 6D).

### Olmesartan blocks MI-induced upregulation of periostin

**Figure 7:**
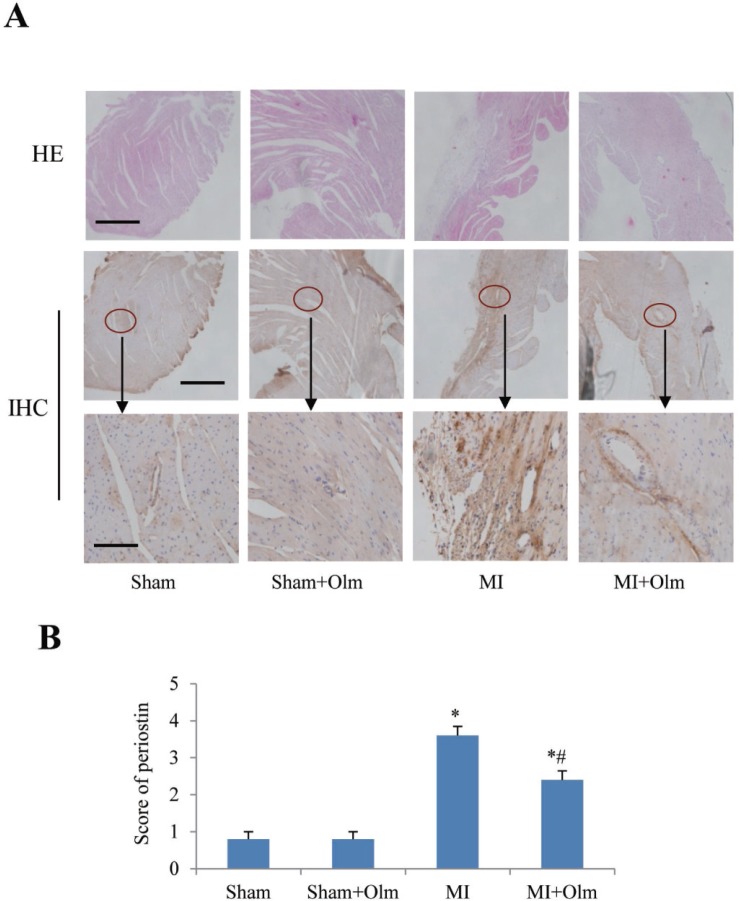
Periostin expression in olmesartan (Olm)-treated mice at 4 weeks after myocardial infarction (MI) (**A**) Hematoxylin-eosin (HE) (top), immunohistochemistry (IHC) of periostin (middle) staining of mouse hearts at 4 weeks after MI. The lower panels show magnifications of the region indicated by black arrows in the upper panels. The scale bar is 200 μm in the top and middle panels, and 100 μm in the lower panel. (**B**) Semi-quantitative analysis of periostin staining in each group. ^*^P < 0.05 vs. their corresponding sham group, ^#^*P <* 0.05 vs. MI group.

Immunohistochemistry staining revealed that periostin expression in the infarcted area was increased 4 weeks after MI in comparison with sham group (Figure 7A, 7B), while treatment of olmesartan significantly blocked the MI-induced upregulation of periostin (Figure 7A, 7B).

### Periostin knockout increases GSK3β and decreases cyclin D1

**Figure 8:**
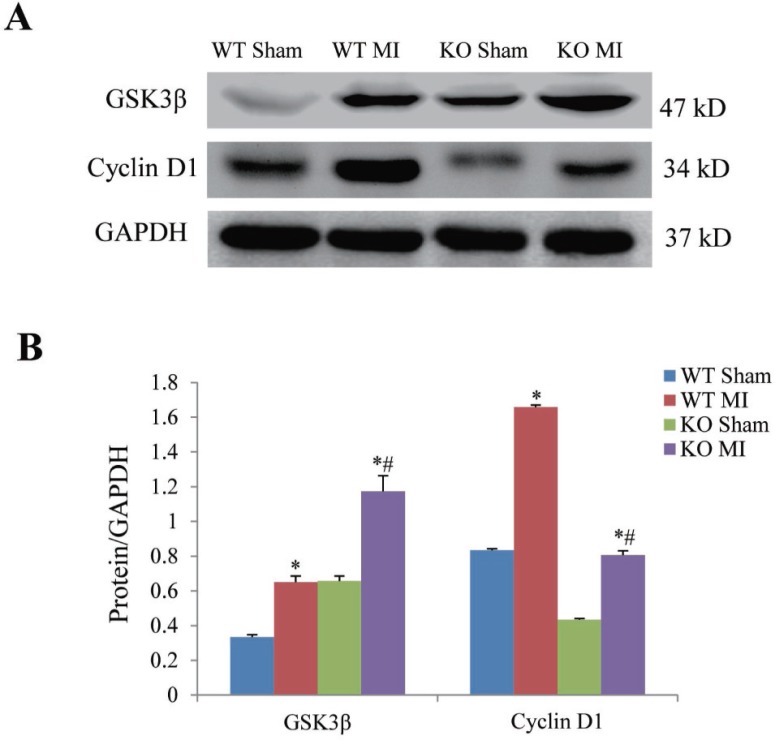
Effect of periostin knockout on expression of GSK3β and cyclin D1 at 7 days after myocardial infarction (**A**) Representative examples of western blotting of myocardial GSK3β and cyclin D1. (**B**) Semi-quantitation of myocardial GSK3β and cyclin D1. ^*^P < 0.05 vs. their corresponding sham group, ^#^*P <* 0.05 vs. MI group, *n* = 5 in each group.

At 7 days after MI, we examined whether periostin was involved in GSK3β-cyclin D1 signal pathway which is believed to play a critical role in fibrosis. We noted that GSK3β protein expression was higher while cyclin D1 protein was significantly lower in the periostin knockout mice than in wildtype mice (Figure 8A and 8B).

## DISCUSSION

In this study, we showed that temocapril or olmesartan or their combination exerted potent inhibitory effect on cardiac remodeling caused by TAC or MI. We assessed the transcriptional effects of these drugs on heart and detected the differences in gene expression in response to effective pharmacological treatments, indicating that these genes may be attributable to the mechanisms induced in cardiac remodeling. More importantly, besides the traditionally well-described genes regulated by ACEI or ARB, such as ACE [19], BNP and ANP [7], our results demonstrated that the molecular effects of ACEI or ARB on cardiac remodeling are associated with the down-regulated expression of EMP1, CARP and periostin, and up-regulated expression of telethonine and tuberin. Global profiling of gene expression in hypertrophic cardiomyopathy has been undertaken in human and animals [12, 20–23], however, few studies have examined the effects of anti-hypertrophic agents on global gene expression pattern in overload pressure-induced cardiac hypertrophy. Some of the altered genes in response to pressure overload found in this study was rarely studied for their role in cardiac hypertrophy. For example no report was found for the role of proteolipid protein 2 in myocardial hypertrophy, and very few reports investigating whether fibulin 2 contributes to cardiac remodeling. Khan *et al.* reported that fibulin-2 plays an essential role in Ang II-induced TGF-β signaling and subsequent myocardial fibrosis [24]. We believe it would be worth to clarify the role of those screened out genes in cardiac remodeling in future studies.

The present study has revealed that TAC-induced cardiac hypertrophy up-regulates 109 genes, and reversion of gene changes in response to treatment with ACEI or ARB supports the idea that these up-regulated genes by TAC are the potential target gene of ACEI or ARB.

The present study revealed that CARP, a cardiac doxorubicin-responsive protein, is up-regulated by TAC. CARP has been identified as a nuclear protein and other investigators confirmed that CARP expression is up-regulated during cardiac hypertrophy *in vivo* [25]. In cultured cardiac myocytes, the CARP promoter is activated by p38MAP kinase which is known as a mediator to several hypertrophic signaling pathways, including angiotensin II. It is also reported that angiotensin II-induced activation of p38 and ERK signaling pathways could be completely inhibited by the AT1 receptor antagonist losartan [26]. Since in our present study ACEI and/or ARB down-regulated the CARP expression, in our present study, CARP may be an important regulator of cardiac hypertrophic process and serve as an indicator to monitoring the pharmacological effects of antihypertrophic agents. Interestingly, our recent studies have demonstrated that CARP promotes cardiomyocyte hypertrophy and apoptosis [27, 28].

Periostin (formally called osteoblast-specific factor 2) is up-regulated by transforming growth factor beta-1 in primary osteoblasts [29]. Additionally, periostin is reported to exist within the embryonic, fetal and adult myocardium [30] and may play a role in extracellular matrix deposition, fibrosis and tissue remodeling following MI [23]. Several studies also revealed that periostin was elevated during cardiac hypertrophy [21, 31]. Our recent study has demonstrated that both cardiomyocytes and fibroblasts expressed periostin and ablation of periostin suppressed post-infarction myocardial regeneration by inhibiting the PI3K/GSK3β/cyclin D1 signaling pathway [32], suggesting a proliferating ability of periostin. Although the mechanisms by which either ACEI or ARB treatment down-regulates periostin remains unclear, the down-regulation of periostin may be one of the mechanisms among the beneficial effects on myocardial remodeling produced by either ACEI or ARB. The finding in this study that olmesartan down-regulated the periostin expression suggests periostin may be an important target gene of ARB, therefore we further investigated the effect of olmesartan on post-MI remodeling and expression of periostin.

We noted that olmesartan attenuated post-MI remodeling partially through downregulating periostin. It’s reported that olmesartan ameliorated atherosclerosis in hyperlipidemic animals and attenuated cardiac remodeling and improved survival in rats with MI, and olmesartan also has renoprotective effects in a remnant kidney model and type 2 diabetic models [33] (a review by Yoshida *et al.*). Our previous study demonstrated that olmesartan prevented cardiac rupture in mice with MI by modulating growth differentiation factor 15 and p53 [34], while Koike *et al.* reported that olmesartan increased the survival rate in rats with MI [35], and Taniyama and his colleagues demonstrated that olmesartan played an anti-fibrotic role by up-regulating hepatocyte growth factor in cardiomyopathic hamsters [36]. These reports are in agreement with our findings that olmesartan is able to attenuate cardiac remodeling. The attenuation of cardiac remodeling by treatment with olmesartan may also be attributable to its inhibition of aldosterone synthesis [37], enhancement of ACE2/Ang(1-7)/Mas axis and suppression of Nox4 expression [38], and a sustained decrease of plasma Ang II [39].

Periostin may play a role in fibrosis and tissue remodeling following myocardial infarction [23, 40]. Several recent studies also revealed that periostin levels were elevated during cardiac hypertrophy [41]. The role of periostin in myocardial fibrosis in adult animals remains controversial [17, 40, 42]. Dennis *et al.* reported that stimulating myocardial regeneration with periostin peptide in large mammals improved post-MI cardiac function but increased myocardial fibrosis [43], while Taniyama *et al.* reported that inhibition of periostin-exon 17 attenuated post-MI fibrosis in adult rats but did not affect cardiomyocyte proliferation [44]. Cho *et al.* demonstrated that injection of mesenchymal stem cells overexpressing periostin into the infarcted regions of rat hearts attenuated post-MI remodeling [45]. Harmandeep *et al.* reported that ablation of periostin-expressing cardiac fibroblast reduced fibrosis without compromising scar stability, and improved cardiac function significantly [46]. These findings support the involvement of periostin in myocardial regeneration and post-MI remodeling in the adult heart but its actual effect seems to be context-dependent.

In conclusion, our findings indicate that olmesartan attenuates cardiac remodeling induced by TAC or MI at least partially through down-regulating periostin, implicating that periostin is a molecular target of ARB for inhibition of cardiac fibrosis.

## METHODS

All procedures were approved by the Animal Care and Use Committee of the Southern Medical University (Guangzhou, Guangdong, China) and were in accordance with the Guide for the Care and Use of Laboratory Animals published by the US National Institutes of Health (NIH Publication, 8th Edition, 2011).

### TAC and MI models and experimental protocols

TAC model. The pressure-overload models were created by TAC in male C57BL/6 mice (8 weeks old, 22–25 g) as we previously described [47]. Briefly, mice were anesthetized with a mixture of ketamine (100 mg/kg i.p) and xylazine (5 mg/kg i.p), and TAC was produced using a 7-0 suture tied twice around the aorta between innominate and left common carotid arteries with a 27-gauge needle. The needle was then gently retracted, yielding a 60–80% constriction of the aorta. To confirm whether the degree of pressure overload is similar between drug-treated and untreated groups, after TAC or sham operation, 3 mice in each group were randomly chosen and used to measure the pressure in ascending aorta using 1.4F Millar catheter at 1 week following surgery, those mice did not receive any drug treatment.

MI model. MI was created by left coronary artery ligation as described elsewhere [48]. In brief, mice were anaesthetized with a mixture of xylazine (5 mg/kg, ip) and ketamine (100 mg/kg, ip), and the depth of anesthesia was assessed by monitoring the pedal withdrawal reflex. Mice were then ventilated and subjected to a left-sided thoracotomy and the left coronary artery ligation to induce MI. Ischemia was judged by myocardial blanching and electrocardiogram ST-segment elevation. Sham operated mice underwent the same procedure without ligation of left coronary artery.

Mice respectively received the vehicle, temocapril (10 mg/kg/day, orally by gavage), olmesartan (10 mg/kg/day, orally by gavage), both temocapril and olmesartan (5 mg/kg/day for both agents, orally by gavage) immediately after TAC or MI. One or 4 weeks later, mice were euthanized to obtain their hearts for analysis of cardiac hypertrophy and gene expression. We decided the dosage of temocapril and olmesartan according to the previous reports from our and other’s laboratories [49, 50]. Our preliminary experiment showed that the mouse could not endure a dose of temocapril 10 mg/kg/d + olmesartan 10 mg/kg/d in the combination therapy group. Both temocapril and olmesartan were provided by Daiichi Sankyo Co., Ltd. (Tokyo, Japan).

Periostin knockout mice (B6;129-Postnt^m1Jmol/J^, Targeted: Null/Knockout, Stock No: 009067. Donated by Jeffery D. Molkentin, Cincinnati Children’s Hospital) were purchased from the Jackson Laboratory (Bar Harbor, ME, USA) [32].

### Triphenyl tetrazolium chloride (TTC) staining

One day after surgery, some mice were killed, their hearts harvested and each heart cut into five pieces. MI was confirmed by staining with 1% TTC (Sigma Aldrich) at 37°C for 20 min. Myocardial infarct size was measured using Image J Analysis software (National Institutes of Health, Bethesda, MD, USA).

### Echocardiographic analysis

Transthoracic echocardiography was performed with a Sonos 4500 and a 15-6 L MHz transducer (Philips, the Netherlands) or a Sequoia 512 system with a 17L-5 probes (Siemens, Germany). Mice were weighed, anesthetized with 2.5% avertin (0.06 ml/10 g), and settled in the left decubitus or supine position. Good two-dimensional short-axis views of the left ventricle were obtained for guided M-mode measurements of the left ventricular posterior wall thickness (LVPWd), left ventricular end-diastolic diameter (LVEDd), and left ventricular end-systolic diameter (LVESd). Left ventricular fractional shortening (LVFS) was calculated: LVFS = (LVEDd–LVESd)/LVEDd × 100.

### RNA preparation and hybridization to oligonucleotide arrays

Total RNA was isolated from whole hearts using TRIzol reagent (GIBCO/BRL) as described by the manufacturer. After synthesizing double-stranded cDNA from the total RNA, an *in vitro* transcription reaction was done to produce biotin-labeled cRNA from cDNA, and the cRNA was fragmented before hybridization. Hybridization, probe washing, staining and probe array scanning were performed following the protocols provided by Affymetrix.

### Polymerase chain reaction analysis (PCR)

Primers were designed using Gene Express software. Total RNA of homogenized murine whole heart or cultured cardiomyocytes was isolated using Total RNA Kit II (Omega) according to the protocol provided by the manufacturer. Using 50 ng /µl of total RNA as a template, a quantitative measurement of gene expression was performed with an ABI Prism 7700 sequence detection system. Quantitect SYBR Green RT-PCR kit (QIAGEN) was used to perform amplifications with One-step protocol as described by the manufacturer. To standardize the quantity of the selected genes, glyceraldehyde-3-phosphate dehydrogenase (GAPDH) was used as the endogenous control since our microarray analysis showed that the level of GAPDH was stable and no significant difference was noted among all the groups. Conventional PCR for periostin and cardiac ankyrin repeat protein (CARP) was also performed.

### Invasive assessment of LV hemodynamics

LV hemodynamics was evaluated before sacrifice of the animals as described elsewhere [51]. Briefly, mice from each group were anesthetized with a combination of xylazine and ketamine (light anesthesia for MI mice), and were ventilated. A Millar catheter was inserted via the right carotid artery and carefully introduced into the LV to measure the heart rate (HR), systolic pressure (LVSP), end diastolic pressure (LVEDP), maximum and minimum rates of change in the LV pressure (dp/dt max and dp/dt min, respectively). Both the contractility index (dp/dt max divided by the pressure at the time of max dp/dt) and the exponential time constant of relaxation (tau) were calculated using a software program (Blood Pressure Module).

### Histological examinations

Heart tissue from different groups was excised, rinsed with phosphate buffered saline (PBS), fixed in 4% paraformaldehyde, embedded in paraffin and cut into 4–6 mm sections. Masson’s trichrome (Azan) staining was used to evaluate myocardial fibrosis. For immunohistochemistry (IHC), after antigen retrieval by incubation in citrate buffer (pH 6.0), sections were incubated with rabbit anti-mouse periostin (Abcam) antibody overnight at 4°C. To assess periostin staining, each tissue section was scanned entirely and its staining intensity would be scored as 0 (negative), 1 (weak), 2 (medium), or 3 (strong). The extent of staining was scored as 0 (0%), 1 (1–25%), 2 (26–50%), 3 (51–75%), or 4 (76–100%), according to the percentages of positively stained areas in relation to those of the whole view field. For each sample, the sum of these two scores was reported as the final staining scores (0–7).

### Cell culture

The neonatal rats were sacrificed by 2% isoflurane inhalation and cervical dislocation. Isolation and culture of ventricular cardiomyocytes was performed as described previously [52]. Cells were cultured for 4 days and then treated with 1 µM Ang II or recombinant adenovirus containing the rat CARP cDNA (Ad-Ankrd1). Cell surface area was also measured by staining with rhodamine phalloidin and diamidino-2-phenylindole dihydrochloride (DAPI). Briefly, the cells were fixed with 4.0% paraformaldehyde in PBS, permeabilized in 0.1% Triton X-100 in PBS, and stained with rhodamine phalloidin (5 mg/ml, Invitrogen) and DAPI (5 mg/mL, Beyotime) by standard immunocytochemical techniques. At least 30 random cells from each group were measured by the Image J software.

Construction of recombinant adenovirus carrying Ankrd1 was performed as described previously [28].

### Western blotting

Proteins were extracted from the cultured cardiomyocytes or the murine heart. Immunoblotting was performed by using antibodies against CARP (Santa Cruz), GSK3β (Santa Cruz), Cyclin D1 (Abcam). Blotting of GAPDH (Santa Cruz) was used as a loading control.

### Data analysis

Affymetrix Murine Genome U74v2A GeneChips were used to evaluate 12,488 genes in this study. GeneSpring 6.0 (Silicon Genetics, Redwood City, CA) was used to perform the analyses. Normalization was done by a combination of 3 steps: rewriting negative values as 0.01, normalizing to the 50th percentile per chip and normalizing to the median per gene. We filtered data using a combination of parameters such as signal confidence (‘present’ flag), fold change (1.5–2), minimum acceptable signal intensity (average difference ≥50 in at least 2 of all groups), and statistical cut-off (*P <* 0.05, student’s *t*-test). Data are presented as means or means ± SEM. One-way ANOVA with Tukey-Kramer exact probability test was used to test the differences among all the groups. *P <* 0.05 was considered statistically significant.

## References

[1] Sakata Y, Nochioka K, Miura M, Takada T, Tadaki S, Miyata S, Shiba N, Shimokawa H (2013). Supplemental benefit of an angiotensin receptor blocker in hypertensive patients with stable heart failure using olmesartan (SUPPORT) trial--rationale and design. J Cardiol.

[2] Hu DY, Huang J, Cai NS, Zhu WL, Li YS, Massaad R, Hanson ME, Dickstein K (2012). A multi-center, double-blind, randomized, parallel group study to evaluate the effects of two different doses of losartan on morbidity and mortality in Chinese patients with symptomatic heart failure intolerant of angiotensin converting enzyme inhibitor treatment. Chin Med J (Engl).

[3] Borghi C, Cosentino ER, Rinaldi ER, Cicero AF (2013). Effect of zofenopril and ramipril on cardiovascular mortality in patients with chronic heart failure. Am J Cardiol.

[4] Nakamura Y, Yoshiyama M, Omura T, Yoshida K, Izumi Y, Takeuchi K, Kim S, Iwao H, Yoshikawa J (2003). Beneficial effects of combination of ACE inhibitor and angiotensin II type 1 receptor blocker on cardiac remodeling in rat myocardial infarction. Cardiovasc Res.

[5] Wu L, Iwai M, Nakagami H, Chen R, Suzuki J, Akishita M, de Gasparo M, Horiuchi M (2002). Effect of angiotensin II type 1 receptor blockade on cardiac remodeling in angiotensin II type 2 receptor null mice. Arterioscler Thromb Vasc Biol.

[6] Mankad S, d’Amato TA, Reichek N, McGregor WE, Lin J, Singh D, Rogers WJ, Kramer CM (2001). Combined angiotensin II receptor antagonism and angiotensin-converting enzyme inhibition further attenuates postinfarction left ventricular remodeling. Circulation.

[7] Kim S, Yoshiyama M, Izumi Y, Kawano H, Kimoto M, Zhan Y, Iwao H (2001). Effects of combination of ACE inhibitor and angiotensin receptor blocker on cardiac remodeling, cardiac function, and survival in rat heart failure. Circulation.

[8] Blaxall BC, Tschannen-Moran BM, Milano CA, Koch WJ (2003). Differential gene expression and genomic patient stratification following left ventricular assist device support. J Am Coll Cardiol.

[9] Lowes BD, Gilbert EM, Abraham WT, Minobe WA, Larrabee P, Ferguson D, Wolfel EE, Lindenfeld J, Tsvetkova T, Robertson AD, Quaife RA, Bristow MR (2002). Myocardial gene expression in dilated cardiomyopathy treated with beta-blocking agents. N Engl J Med.

[10] Nagata K, Somura F, Obata K, Odashima M, Izawa H, Ichihara S, Nagasaka T, Iwase M, Yamada Y, Nakashima N, Yokota M (2002). AT1 receptor blockade reduces cardiac calcineurin activity in hypertensive rats. Hypertension.

[11] Sandmann S, Yu M, Unger T (2001). Transcriptional and translational regulation of calpain in the rat heart after myocardial infarction—effects of AT and AT receptor antagonists and ACE inhibitor. Br J Pharmacol.

[12] Friddle CJ, Koga T, Rubin EM, Bristow J (2000). Expression profiling reveals distinct sets of genes altered during induction and regression of cardiac hypertrophy. Proc Natl Acad Sci U S A.

[13] Segers VF, Lee RT (2010). Protein therapeutics for cardiac regeneration after myocardial infarction. J Cardiovasc Transl Res.

[14] Katsuragi N, Morishita R, Nakamura N, Ochiai T, Taniyama Y, Hasegawa Y, Kawashima K, Kaneda Y, Ogihara T, Sugimura K (2004). Periostin as a novel factor responsible for ventricular dilation. Circulation.

[15] O’Meara CC, Wamstad JA, Gladstone RA, Fomovsky GM, Butty VL, Shrikumar A, Gannon JB, Boyer LA, Lee RT (2015). Transcriptional Reversion of Cardiac Myocyte Fate During Mammalian Cardiac Regeneration. Circ Res.

[16] Kudo A (2011). Periostin in fibrillogenesis for tissue regeneration: periostin actions inside and outside the cell. Cell Mol Life Sci.

[17] Shimazaki M, Nakamura K, Kii I, Kashima T, Amizuka N, Li M, Saito M, Fukuda K, Nishiyama T, Kitajima S, Saga Y, Fukayama M, Sata M (2008). Periostin is essential for cardiac healing after acute myocardial infarction. J Exp Med.

[18] Sozmen M, Devrim AK, Kabak YB, Devrim T, Sudagidan M (2017 Sep 11). The effects of periostin in a rat model of isoproterenol: mediated cardiotoxicity. Cardiovasc Toxicol.

[19] Walther T, Schubert A, Falk V, Binner C, Walther C, Doll N, Fabricius A, Dhein S, Gummert J, Mohr FW (2002). Left ventricular reverse remodeling after surgical therapy for aortic stenosis: correlation to Renin-Angiotensin system gene expression. Circulation.

[20] Lim DS, Roberts R, Marian AJ (2001). Expression profiling of cardiac genes in human hypertrophic cardiomyopathy: insight into the pathogenesis of phenotypes. J Am Coll Cardiol.

[21] Aronow BJ, Toyokawa T, Canning A, Haghighi K, Delling U, Kranias E, Molkentin JD, Dorn GW (2001). Divergent transcriptional responses to independent genetic causes of cardiac hypertrophy. Physiol Genomics.

[22] Liu T, Lai H, Wu W, Chinn S, Wang PH (2001). Developing a strategy to define the effects of insulin-like growth factor-1 on gene expression profile in cardiomyocytes. Circ Res.

[23] Johnatty SE, Dyck JR, Michael LH, Olson EN, Abdellatif M (2000). Identification of genes regulated during mechanical load-induced cardiac hypertrophy. J Mol Cell Cardiol.

[24] Khan SA, Dong H, Joyce J, Sasaki T, Chu ML, Tsuda T (2016). Fibulin-2 is essential for angiotensin II-induced myocardial fibrosis mediated by transforming growth factor (TGF)-beta. Lab Invest.

[25] Aihara Y, Kurabayashi M, Saito Y, Ohyama Y, Tanaka T, Takeda S, Tomaru K, Sekiguchi K, Arai M, Nakamura T, Nagai R (2000). Cardiac ankyrin repeat protein is a novel marker of cardiac hypertrophy: role of M-CAT element within the promoter. Hypertension.

[26] Polontchouk L, Ebelt B, Jackels M, Dhein S (2002). Chronic effects of endothelin 1 and angiotensin II on gap junctions and intercellular communication in cardiac cells. Faseb J.

[27] Shen L, Chen C, Wei X, Li X, Luo G, Zhang J, Bin J, Huang X, Cao S, Li G, Liao Y (2015). Overexpression of ankyrin repeat domain 1 enhances cardiomyocyte apoptosis by promoting p53 activation and mitochondrial dysfunction in rodents. Clin Sci (Lond).

[28] Chen C, Shen L, Cao S, Li X, Xuan W, Zhang J, Huang X, Bin J, Xu D, Li G, Kitakaze M, Liao Y (2014). Cytosolic CARP promotes angiotensin II- or pressure overload-induced cardiomyocyte hypertrophy through calcineurin accumulation. PLoS One.

[29] Horiuchi K, Amizuka N, Takeshita S, Takamatsu H, Katsuura M, Ozawa H, Toyama Y, Bonewald LF, Kudo A (1999). Identification and characterization of a novel protein, periostin, with restricted expression to periosteum and periodontal ligament and increased expression by transforming growth factor beta. J Bone Miner Res.

[30] Kruzynska-Frejtag A, Machnicki M, Rogers R, Markwald RR, Conway SJ (2001). Periostin (an osteoblast-specific factor) is expressed within the embryonic mouse heart during valve formation. Mech Dev.

[31] Stanton LW, Garrard LJ, Damm D, Garrick BL, Lam A, Kapoun AM, Zheng Q, Protter AA, Schreiner GF, White RT (2000). Altered patterns of gene expression in response to myocardial infarction. Circ Res.

[32] Chen Z, Xie J, Hao H, Lin H, Wang L, Zhang Y, Chen L, Cao S, Huang X, Liao W, Bin J, Liao Y (2017). Ablation of periostin inhibits post-infarction myocardial regeneration in neonatal mice mediated by the phosphatidylinositol 3 kinase/glycogen synthase kinase 3beta/cyclin D1 signalling pathway. Cardiovasc Res.

[33] Yoshida K, Kohzuki M (2004). Clinical and experimental aspects of olmesartan medoxomil, a new angiotensin II receptor antagonist. Cardiovasc Drug Rev.

[34] Chen B, Lu D, Fu Y, Zhang J, Huang X, Cao S, Xu D, Bin J, Kitakaze M, Huang Q, Liao Y (2014). Olmesartan prevents cardiac rupture in mice with myocardial infarction by modulating growth differentiation factor 15 and p53. Br J Pharmacol.

[35] Koike H, Sada T, Mizuno M (2001). *In vitro* and *in vivo* pharmacology of olmesartan medoxomil, an angiotensin II type AT1 receptor antagonist. J Hypertens Suppl.

[36] Taniyama Y, Morishita R, Nakagami H, Moriguchi A, Sakonjo H, Shokei K, Matsumoto K, Nakamura T, Higaki J, Ogihara T (2000). Potential contribution of a novel antifibrotic factor, hepatocyte growth factor, to prevention of myocardial fibrosis by angiotensin II blockade in cardiomyopathic hamsters. Circulation.

[37] Miura S, Nakayama A, Tomita S, Matsuo Y, Suematsu Y, Saku K (2015). Comparison of aldosterone synthesis in adrenal cells, effect of various AT1 receptor blockers with or without atrial natriuretic peptide. Clin Exp Hypertens.

[38] Tanno T, Tomita H, Narita I, Kinjo T, Nishizaki K, Ichikawa H, Kimura Y, Tanaka M, Osanai T, Okumura K (2016). Olmesartan Inhibits Cardiac Hypertrophy in Mice Overexpressing Renin Independently of Blood Pressure: Its Beneficial Effects on ACE2/Ang(1-7)/Mas Axis and NADPH Oxidase Expression. J Cardiovasc Pharmacol.

[39] Tsutamoto T, Nishiyama K, Yamaji M, Kawahara C, Fujii M, Yamamoto T, Horie M (2010). Comparison of the long-term effects of candesartan and olmesartan on plasma angiotensin II and left ventricular mass index in patients with hypertension. Hypertens Res.

[40] Li L, Fan D, Wang C, Wang JY, Cui XB, Wu D, Zhou Y, Wu LL (2011). Angiotensin II increases periostin expression via Ras/p38 MAPK/CREB and ERK1/2/TGF-beta1 pathways in cardiac fibroblasts. Cardiovasc Res.

[41] Teekakirikul P, Eminaga S, Toka O, Alcalai R, Wang L, Wakimoto H, Nayor M, Konno T, Gorham JM, Wolf CM, Kim JB, Schmitt JP, Molkentin JD (2010). Cardiac fibrosis in mice with hypertrophic cardiomyopathy is mediated by non-myocyte proliferation and requires Tgf-beta. J Clin Invest.

[42] Kuhn B, del Monte F, Hajjar RJ, Chang YS, Lebeche D, Arab S, Keating MT (2007). Periostin induces proliferation of differentiated cardiomyocytes and promotes cardiac repair. Nat Med.

[43] Ladage D, Yaniz-Galende E, Rapti K, Ishikawa K, Tilemann L, Shapiro S, Takewa Y, Muller-Ehmsen J, Schwarz M, Garcia MJ, Sanz J, Hajjar RJ, Kawase Y (2013). Stimulating myocardial regeneration with periostin Peptide in large mammals improves function post-myocardial infarction but increases myocardial fibrosis. PLoS One.

[44] Taniyama Y, Katsuragi N, Sanada F, Azuma J, Iekushi K, Koibuchi N, Okayama K, Ikeda-Iwabu Y, Muratsu J, Otsu R, Rakugi H, Morishita R (2016). Selective Blockade of Periostin Exon 17 Preserves Cardiac Performance in Acute Myocardial Infarction. Hypertension.

[45] Cho YH, Cha MJ, Song BW, Kim IK, Song H, Chang W, Lim S, Ham O, Lee SY, Choi E, Kwon HM, Hwang KC (2012). Enhancement of MSC adhesion and therapeutic efficiency in ischemic heart using lentivirus delivery with periostin. Biomaterials.

[46] Kaur H, Takefuji M, Ngai CY, Carvalho J, Bayer J, Wietelmann A, Poetsch A, Hoelper S, Conway SJ, Möllmann H, Looso M, Troidl C, Offermanns S, Wettschureck N (2016). Targeted Ablation of Periostin-Expressing Activated Fibroblasts Prevents Adverse Cardiac Remodeling in Mice. Circ Res.

[47] Liao Y, Ishikura F, Beppu S, Asakura M, Takashima S, Asanuma H, Sanada S, Kim J, Ogita H, Kuzuya T, Node K, Kitakaze M, Hori M (2002). Echocardiographic assessment of LV hypertrophy and function in aortic-banded mice: necropsy validation. Am J Physiol Heart Circ Physiol.

[48] Xuan W, Wu B, Chen C, Chen B, Zhang W, Xu D, Bin J, Liao Y (2012). Resveratrol improves myocardial ischemia and ischemic heart failure in mice by antagonizing the detrimental effects of fractalkine. Crit Care Med.

[49] Takemoto M, Egashira K, Tomita H, Usui M, Okamoto H, Kitabatake A, Shimokawa H, Sueishi K, Takeshita A (1997). Chronic angiotensin-converting enzyme inhibition and angiotensin II type 1 receptor blockade: effects on cardiovascular remodeling in rats induced by the long-term blockade of nitric oxide synthesis. Hypertension.

[50] Sanada S, Kitakaze M, Node K, Takashima S, Ogai A, Asanuma H, Sakata Y, Asakura M, Ogita H, Liao Y, Fukushima T, Yamada J, Minamino T (2001). Differential subcellular actions of ACE inhibitors and AT receptor antagonists on cardiac remodeling induced by chronic inhibition of NO synthesis in rats. Hypertension.

[51] Wei X, Wu B, Zhao J, Zeng Z, Xuan W, Cao S, Huang X, Asakura M, Xu D, Bin J, Kitakaze M, Liao Y (2015). Myocardial Hypertrophic Preconditioning Attenuates Cardiomyocyte Hypertrophy and Slows Progression to Heart Failure Through Upregulation of S100A8/A9. Circulation.

[52] Xie J, Cui K, Hao H, Zhang Y, Lin H, Chen Z, Huang X, Cao S, Liao W, Bin J, Kitakaze M, Liao Y (2016). Acute hyperglycemia suppresses left ventricular diastolic function and inhibits autophagic flux in mice under prohypertrophic stimulation. Cardiovasc Diabetol.

